# Biochar Influences the Transformation and Translocation of Antimony in the Rhizosphere–Rice System

**DOI:** 10.3390/toxics13050389

**Published:** 2025-05-13

**Authors:** Qiuxiang Huang, Fande Meng, Wenzhe Chen, Yongbing Cai, Enzong Xiao

**Affiliations:** 1 College of Chemistry and Materials Engineering, Anhui Science and Technology University, Bengbu 233030, China; huangqx@ahstu.edu.cn; 2 Anhui Province Quartzs and Purification and Photovoltaic Glass Engineering Research Center, Chuzhou 233100, China; 3 College of Resource and Environment, Anhui Science and Technology University, Chuzhou 233100, China; yjs2022312@ahstu.edu.cn (W.C.); caiyb@ahstu.edu.cn (Y.C.); 4 School of Environmental Science and Engineering, Guangzhou University, Guangzhou 510006, China; xiaoez@gzhu.edu.cn

**Keywords:** antimony, rhizosphere, transformation and migration behavior, biochar

## Abstract

The rhizosphere is a crucial interface that connects the soil and the roots of plants, playing a critical role in regulating soil biochemical functions and processes. Biochar, an increasingly common soil amendment, can directly or indirectly affect the redistribution behavior of heavy metal(loid)s. Our study used a rice pot experiment to investigate the redistribution behavior of antimony (Sb) in the rhizosphere–rice system during the four key rice growth stages and analyze the effects of biochar (BC). Biochar increased pH, soil organic matter (SOM), and dissolved organic carbon (DOC) but decreased Eh, affecting Sb redistribution in the rhizosphere–rice system. The Sb fractions were altered with rice growth and the addition of BC. For example, bioavailable Sb increased by 1.57–32.97% in the presence of BC across all rice growth stages. Biochar reduced the BCF and TFR-S of Sb but elevated the TFS-G, indicating that biochar reduced Sb migration from the soil to the rice roots and the rice roots to shoots but increased Sb migration from rice shoots to grains. This study highlights the potential use of biochar as a reclamation agent in remediating Sb-contaminated soils and protecting human health from Sb through the food chain.

## 1. Introduction

Soil is the foundation of agricultural production and is crucial for ensuring food safety. As industrialization advances, large quantities of chemical agents are introduced into soil ecosystems, leading to soil contamination, which has emerged as a critical issue garnering increasing attention from policymakers, researchers, and the public [1,2,3]. Heavy metals (HMs) are the predominant pollutants in soil environments, exhibiting chemical fraction polymorphism, resistance to degradation, and a significant propensity for bioaccumulation, threatening food safety, agricultural sustainability, and public health [1,3]. Antimony (Sb) is an extensively used metallic element in fire retardants, glass decolorizers, and automotive braking pads, with China being the world’s leading producer, and the pollution induced by Sb warrants significant concern in China, especially around Sb mining and smelting areas and near traffic routes [2,4,5]. Sb entering the human body via various pathways, including the food chain, may cause substantial detrimental effects on human health at high concentrations [6]. The primary sources of Sb in soil environments are anthropogenic activities, such as smelting, fossil fuel combustion, mining, and vehicle braking [2,6]. Sb enters the soil and establishes a new equilibrium through multiple geochemistry processes, including adsorption–desorption and oxidation–reduction [2]. However, changes in soil environmental factors (EFs), especially in the rhizosphere, which vary with plant growth, can easily affect the equilibrium status [7].

Rhizosphere soil is a thin layer that closely adheres to the plant root surface, ranging from a few millimeters to a few centimeters in thickness, and is primarily shaped and driven by root exudates [8,9]. It is the critical interface via which plants interact with nutrient elements, microorganisms, organic matter, and pollutants, and it plays a vital role in food security and ecosystem regulation [9,10,11]. Meier et al. documented that the rhizosphere releases approximately 40% of total nitrogen (TN) and 50% of total carbon (TC) in soil environments, making it crucial for managing global nitrogen and carbon cycles [12]. In addition, EFs in the rhizosphere differ significantly from those in the bulk soil. Previous results showed that the rhizosphere pH shifted towards neutral with plant growth [13], and the levels of low-molecular-weight organic matter, carbon, nitrogen, phosphorus, microbial biomass, and bioavailable iron were substantially higher in the rhizosphere than in the bulk soil [14,15,16]. In addition to being influenced by plant growth at the roots, the rhizosphere is also susceptible to soil additives like biochar [17,18].

Biochar is a widely used soil amendment with a high carbon content, porosity rate, surface area, and environmental resistance, as well as acidic functional groups [19]. It can regulate ecological variables, such as pH, Eh, SOM, and DOM, to influence the redistribution of HMs in the soil–plant system. For example, Ibrahim et al. reported that biochar improved soil physical and chemical properties while decreasing HM bioaccumulation in summer squash [20]. Furthermore, biochar can indirectly regulate HM redistribution by altering the microbial community, as demonstrated by Hua et al., who showed that biochar influences the microbial community and mediates the redistribution of Sb fractions [21].

Although many papers have documented the use of biochar as an amendment in soil enhancement for crop production, soil contamination remediation, and rhizosphere soil modulation [18,22,23], and the impacts of biochar on soil EFs to influence the redistribution of Sb in the soil–plant system [21,24,25], few have investigated the effects of biochar on Sb redistribution behavior in the rhizosphere–rice system. The present study employed biochar produced from Paulownia bio-waste (*Paulownia fortune*) in rice pot experiments to investigate its impact on Sb redistribution behavior and to evaluate the regulatory mechanisms within the rhizosphere–rice system.

## 2. Materials and Methods

### 2.1. Chemical Reagents and Materials

Guaranteed reagents (GRs) HCl, H_2_SO_4_, HNO_3_, and K_2_Cr_2_O_7_, analytical reagents (ARs) CaCl_2_, MgCl_2_, CH_3_COOH, CH_3_COONa, NH_2_OH·HCl, H_2_O_2_, CH_3_COONH_4_, NaOH, and HF, and spectroscopic-purity reagent KBr were purchased from Sinopharm Group Chemical Reagent Co., Ltd., Shanghai, China. Rice seeds of Nanjing 46 were purchased from the Yichen Seed Industry in Nanjing, China.

### 2.2. Soil Sampling, Biochar Preparation, and Subsequent Characterization

Soil samples were collected from the soil surface layer (0–20 cm) of a paddy field adjacent to an Sb-Au mining site by the method of random sampling in August 2022. The soil was contaminated by the breach of the tailings dam in 2010, located in Dongzhi County, Chizhou, Anhui Province, China (30°32′53″ N, 116°44′52″ E), as detailed by Meng et al. [26]. After pretreatment, the soil samples were air-dried, sieved through a 2 mm mesh, thoroughly mixed, and evaluated for basic physicochemical properties. A pH meter (FE22-Standard, Mettler Toledo, Greifensee, Switzerland) was used to determine the pH of soil–water mixes at a 1:5 (g:mL) ratio. Soil organic matter (SOM) was analyzed using the commonly employed hydrated heat K_2_Cr_2_O_7_ oxidation colorimetry method [27]. The soil particles were analyzed using a Masterzizer 2000 (Malvern, UK). Furthermore, the total Sb content was determined using an inductively coupled plasma optical emission spectrometer (ICP-OES) (Avio 200, PerkinElmer, Waltham, MA, USA), as described in Meng et al. [26].

The biochar was derived from *Paulownia* bio-waste, a widely distributed and fast-growing species with high biomass yield in China, using a water–fire coupled process [28], henceforth abbreviated as BC. The acquired BC was dried in an oven at 85 °C until it achieved a constant weight, and then ground and sieved through a 0.25 mm mesh. Subsequently, we examined the properties, including (1) the pH of solid–water mixtures at a 1:10 (g:mL) ratio utilizing a pH meter (FE22-Standard, Mettler Toledo, Switzerland), (2) the carbon content via an elemental analyzer (Vario Micro cube, Elementar, Langenselbold, Germany), (3) the ash content determined by mass loss before and after exposure to 800 °C for 4 h in a muffle furnace (SXG18123, Tianjin, China), (4) characterization of functional groups through Fourier transform infrared spectroscopy (FTIR) (Nicolet iS50, Thermo Fisher Scientific, Waltham, MA, USA), (5) quantification of acidic functional groups using the titration method as per the International Humic Substances Society (IHSS) [29], and (6) assessment of surface morphology using scanning electron microscopy (SEM) (S-4800, Hitachi, Tokyo, Japan).

### 2.3. Pot Experiments

A 2 cm thick layer of small stones with diameters ranging from 2 mm to 2 cm was placed in a 30 cm diameter plexiglass column. The stone layer was then covered with 30 cm of soil sample, either unamended or amended with 3% biochar by weight, indicated as S1 and S1-BC, respectively. After 7 days of waterlogging, 25–30 rice seeds were sown in the soil. Throughout the rice growth stages, the soil water content in the plexiglass column was maintained between 25% and 60% by adding deionized water, as determined by a TMS-4 logger (TOMST, Praha, Czech Republic). Hoagland solution was used to deliver essential nutrients during rice growth. Rhizosphere soil and plant samples were collected at four critical rice growth stages: tillering (TIL), jointing (JOI), grain filling (GRF), and maturity (MAT). The specific sampling method is described below.

In brief, 3 to 5 rice plants with comparable growing conditions were manually pulled out from the soil to prepare rhizosphere soil and plant samples. The uprooted rice plants were placed in a plastic container and gently shaken to dislodge the loosely attached soil from the rice root. The plants were then set on clean aluminum foil and air-dried for 24 h, and finally, the air-dried rice root was placed in a plastic container to repeat the shaking process to collect the rhizosphere soil, as described by Gao et al. [30]. After the collection of the rhizosphere soil, the rice plants were washed three times with deionized water before being separated into shoots and roots at the TIL, JOI, and GRF growth stages and then into shoots, roots, and grains at the MAT stage. The roots and grains were dried at 85 °C to a constant weight, while the shoots were fixed at 105 °C for 30 min before being dried at 85 °C to a constant weight. All treatments were performed in triplicate, with the unplanted soil cylinders serving as controls, labeled as CK (unamended with biochar) and CK-BC (amended with 3% biochar by weight).

### 2.4. Sample Analysis

#### 2.4.1. Environmental Factors of Rhizosphere Soil

After pretreatment of the rhizosphere, the environmental factors (EFs) were assessed as follows: (1) pH was determined using a pH meter for a soil–water mixture at a 1:5 ratio (g:mL) (FE22-Standard, Mettler Toledo, Switzerland); (2) soil organic matter (SOM) was evaluated via the traditional method of hydrated heat K_2_Cr_2_O_7_ oxidation colorimetry [27]; (3) electrochemical potential (Eh) was directly measured with an oxidation–reduction potential meter (HED-QX6550, Horde Elextric, Weifang, China); and (4) dissolved organic matter (DOC) was quantified using a total organic carbon analyzer (TOC-L, Shimadzu, Kyoto, Japan). In brief, the samples were prepared by weighing 0.5 g of soil into a glass container with 10 mL of ultrapure water and placing it in a temperature-controlled shaker set to 120 rpm at room temperature. Before adding the soil, concentrated H_2_SO_4_ was used to remove the organic matter in the glass container. After 24 h, the glass containers with mixtures were centrifuged for 15 min at 4500 rpm, and the solution was passed through a 0.45 μm filter before the analysis.

#### 2.4.2. Bioavailable Sb

The bioavailable Sb was extracted with 0.1 mol/L CaCl_2_, and the content was determined using inductively coupled plasma mass spectrometry (ICP-MS) (Agilent 7800, Agilent, Santa Clara, CA, USA). To begin, we weighed 1 g of rhizosphere soil from the TIL, JOI, GRF, and MAT stages into a 15 mL centrifuge tube with 10 mL of 0.1 mol/L CaCl_2_ and placed the sealed centrifuges in a temperature-controlled shaker at 120 rpm under 25 °C. After 24 h, we centrifuged the tubes at 4500 rpm for 15 min and filtered the solution through a 0.45 μm membrane before analysis with ICP-MS (Agilent 7800, Agilent, USA).

#### 2.4.3. The Sb Fractions

HMs interact with soil components to form diverse bound fractions, reflecting their mobility and bioavailability. The modified Tessier sequential extraction method was utilized for acquiring the Sb fractions, with the HNO_3_-HCl-HF substituted by aqua regia (v(HNO_3_):v(HCl) = 1:3) to extract the residue fraction [31]. The Sb fractions were classified into five categories, namely exchangeable (EXC), carbonate-bound (CARB), Fe-Mn oxide-bound (Fe-Mn), organic-bound (OM), and residue (RES) fractions, based on the extraction method described by Meng et al. and Zheng et al. [26,31].

#### 2.4.4. Sb Content in Plants

To evaluate Sb bioaccumulation by rice plants and the effects of biochar, we measured the Sb levels in rice shoots, roots, and grains using ICP-MS (Agilent 7800, Agilent Technologies, USA). In brief, 0.1 g dried shoot, root, or grain was weighed into a polytetrafluoroethylene (PTFE) tube containing 0.5 mL HF, 2 mL concentrated HNO_3_, and 1 mL H_2_O_2_. The tubes were placed on a heating plate for 2 h at 120 °C. The digestion protocol was repeated several times to ensure the complete digestion of the plant sample. After naturally cooling to room temperature, the contents of the PTFE tube were transferred to a 25 mL glass flask and brought to the calibration mark with ultrapure water. The solutions were filtered through a 0.22 μm membrane filter, and the Sb content was determined using ICP-MS.

The bioconcentration factor (BCF) and translocation factor (TF) were computed to evaluate Sb’s migratory potential from the soil to the roots, shoots, and grains in rice [32]. The calculations were performed using the following equations:

BCF = C_R_/C_RS_ (1)

TF_S-G_ = C_G_/C_S_ (2)

TF_R-S_ = C_S_/C_R_ (3)

where C_R_, C_S_, C_G_, and C_RS_ represent Sb concentrations in rice roots, shoots, grains, and rhizosphere soil, respectively.

### 2.5. Statistical Analysis and Graphical Process

Data calculations, analysis, and graphical processing were performed using Microsoft 2010 and Origin 2024 (OriginLab, Northampton, MA, USA).

## 3. Results and Discussion

### 3.1. Properties of Soil and Biochar

Our study analyzed the soil and biochar (BC) properties. The results showed that the soil sample had a low pH of 5.88 and organic carbon of 1.01%, belonging to silty loam with a high silt content of 61.8% and sand content of 22.4%, impeding the immobilization of HMs in the soil [33,34,35,36]. The Sb content was 94.41 mg/kg, and the bioavailable Sb was 2.21 mg/kg. The contaminant factor (CF), which reflects the degree of contamination in soil, was 38.35 (>5), falling into the high contamination degree category. The degree of soil contamination was calculated using the Sb concentration and the maximum Sb concentration allowed in the soil [37]. The BC derived from Paulownia bio-waste using a water–fire coupled method had a slightly alkaline pH (7.98) and high carbon content (69.58%), ash content (20.85%), and surface area (85.70 m^2^/g). The SEM results demonstrated high porosity in the biochar (Figure S1a), whereas the FTIR spectrum revealed biochar with multiple functional groups (including –COOH, –OH, and –NH_2_) (Figure S1b). The carboxyl and phenolic hydroxyl groups had concentrations of 0.46 mol/kg and 0.15 mol/kg, respectively, and could interact with HMs and lower their mobility [19].

### 3.2. Variations in Environmental Factors in Rhizosphere

**Figure 1:**
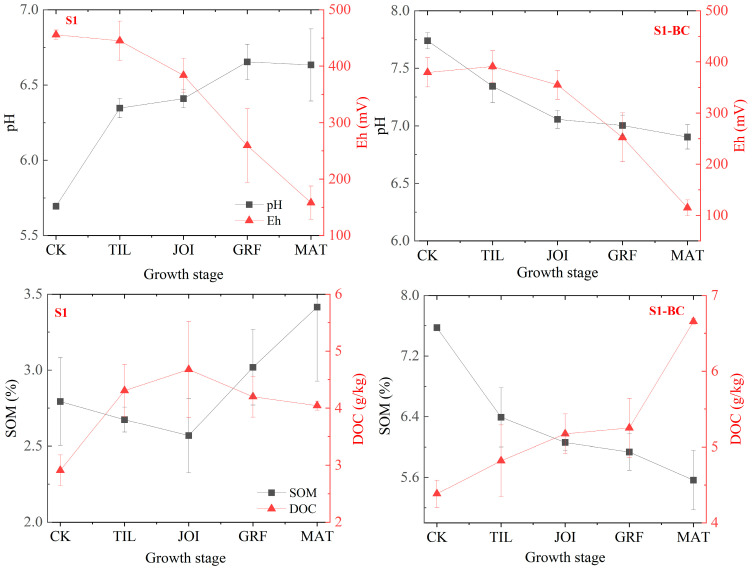
Variations in pH, Eh, SOM, and DOC during rice growth (S1: soil; S1-BC: soil with biochar).

The EFs of pH, Eh, SOM, and DOC play key roles in regulating the redistribution of Sb fractions in soil [24,38]. The results revealed that the pH, Eh, SOM, and DOC varied with rice growing and were influenced by BC (Figure 1).

In S1, the pH increased from 5.70 to 6.63 during the rice growth cycle, a 16.32% increase, primarily due to the neutralizing effects of root exudates and the rise in SOM with multiple functional groups, which consumed acidic substances [39,40]. Hong et al. obtained similar results, where the soil pH increased in relatively acidic soil due to the plant roots [13]. Conversely, the Eh value decreased from 456 mV to 158 mV, a 63.35% reduction, indicating that the rhizosphere environment changed from an oxidation to a reduction condition with rice growth, primarily due to a rise in pH and SOM, as well as the waterlogging conditions [35,41]. From the TIL to MAT stages, SOM showed a decreasing–increasing trend. From the TIL to JOI stages, SOM decreased from 2.79% to 2.56%, while at the MAT stage, it increased to 3.41%. This increase may be due to the availability of suitable growing conditions for rice, which include a pH increase, moderate temperature, and moisture, which caused SOM to decompose at the early growth stage and the organic matter input to surpass the decomposition process at the late growth stage [42,43,44]. The soil moisture was displayed in Figure S2. DOC showed an adverse trend to SOM, with the DOC content increasing from 2.91 g/kg to 4.68 g/kg between the rice TIL and JOI stages and then decreasing to 4.04 g/kg from the JOI to MAT stages, primarily due to the comprehensive utilization of DOC, pH increase, and input and SOM decomposition varying with rice growth [43,44].

Adding biochar significantly affected the EFs of pH, Eh, SOM, and DOC, increasing pH from 5.70 to 7.74, SOM content from 2.79% to 7.57%, and DOC content from 2.91 g/kg to 4.38 g/kg, but decreasing Eh values from 455.85 mV to 379.28 mV, owing to the biochar’s introduction of alkaline materials, dissolved organic matter, and organic matter into the soil [45]. The pH, Eh, SOM, and DOC in S1-BC varied across the rice growth stages. The pH showed an opposite trend to S1 in that it dropped to 6.90 during the rice growth cycle, a 10.85% reduction, owing to the combined impacts of alkaline material consumption by acid functional groups of organic acids secreted from the roots, SOM decomposition, and variable waterlogging conditions [40]. Hong et al. showed that the soil pH decreased with plant growth [13]. The Eh showed a similar trend to pH in that it decreased from 379.28 mV to 114.85 mV, a reduction of 2.73–27.46% compared to S1. Adding BC increased the pH, with the SOM content being the primary contributor [35,41]. SOM showed a different variation to S1 in that the content decreased from 7.57% to 5.56% across all rice growth stages, primarily due to the biochar addition, which improved the soil environment for the microbial community and promoted the decomposition of SOM [46]. Furthermore, we discovered that during the entire rice growing period, which lasted roughly five months, the decline in SOM was only 26.55%, indicating that biochar has a high level of environmental resistance in soil environments [19]. DOC showed a negative correlation with SOM, increasing from 4.38 g/kg to 6.66 g/kg during rice growth, primarily due to the high pH, low Eh, introduction of DOC from BC, and decomposition of SOM by microbial communities and the pH decrease [46,47].

These results indicate that EFs such as pH, Eh, SOM, and DOC varied across rice growth stages, and biochar significantly impacted the redistribution of Sb fractions in the rhizosphere–rice system [24,38].

### 3.3. Bioavailable Sb in Rhizosphere

The bioavailable fraction of HMs is important for assessing their potential for mobility and toxicity to human health. Multiple EFs influence the bioavailability of HMs in soil, including pH, Eh, SOM, and DOC [38,48].

**Figure 2:**
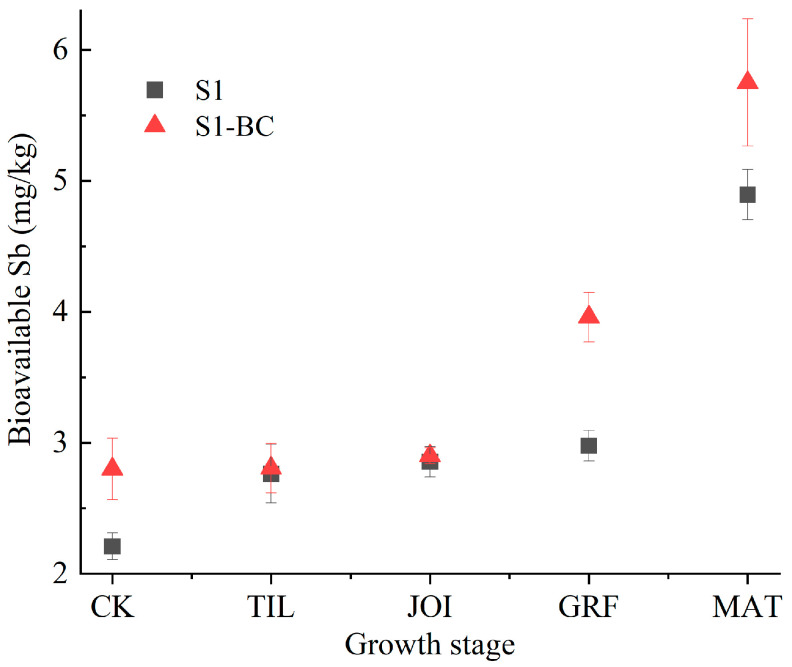
Variations in bioavailable Sb in S1 and S1-BC (S1: soil; S1-BC: soil with biochar).

The bioavailable Sb was altered dynamically with rice growth and BC addition (Figure 2). In S1, the bioavailable Sb concentration increased from 2.21 mg/kg to 4.90 mg/kg during rice growth, primarily due to the soil’s overall shifts from an acidic to a neutral state, and from an oxidation to a reduction state, and a rise in DOC [49,50]. In S1-BC, the variation in bioavailable Sb followed a similar trend as in S1, increasing from 2.80 mg/kg to 5.75 mg/kg, a rise of 1.57–32.97% in the presence of BC, indicating that biochar boosted the bioavailable Sb in the rhizosphere soil as adding BC increased the pH and DOC content but decreased Eh [49,50]. In general, DOC derived from BC with abundant functional groups interacts with Sb through complexation, chelation, and adsorption, thereby increasing its bioavailability [51,52]. Thus, we conclude that the bioavailable Sb varied with the rice growth, and adding BC increased the bioavailable Sb concentrations.

### 3.4. Redistribution of Sb Fractions in Rhizosphere

**Figure 3:**
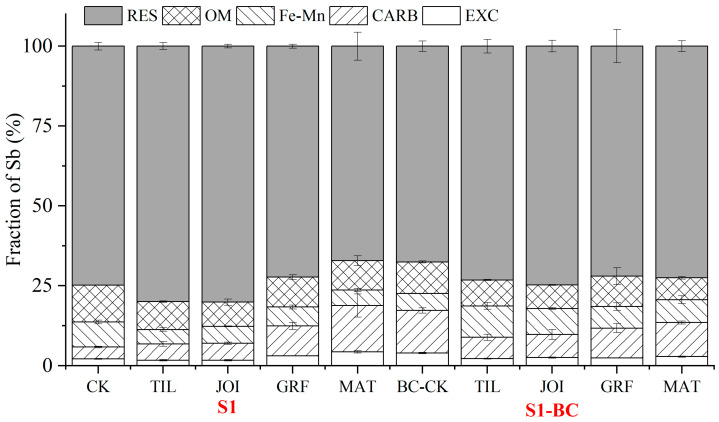
The redistribution of Sb fractions in the rhizosphere (S1: soil; S1-BC: soil with biochar).

The HM fractions were used to reflect the bioavailability and mobility and to assess their potential risks [7,53]. The results showed that the Sb fractions fluctuated with rice growth and were impacted by BC, as depicted in Figure 3.

In S1, the EXC fraction of Sb grew from 1.71% to 4.29% across all the rice growth stages, a 150.96% increase, primarily due to spikes in pH and DOC and a decrease in Eh [49,50]. The CARB fraction was increased from 5.04% to 14.51%, a 188.17% increase, primarily due to a rise in pH, which boosted carbonate production [54]. The OM fraction decreased from the TIL to JOI stages and subsequently increased until the MAT stage, following the same trend as SOM, suggesting that organic matter influenced the OM fraction of Sb. Diquattro et al. reported similar results [7]. RES decreased from 80.14% to 67.19% with rice growth, a 16.16% reduction, owing primarily to variable EFs that induced RES fraction transfer into other factions [7,49,50,54].

The BC addition significantly affected the rhizosphere EFs, boosting pH, DOC, and SOM while reducing Eh, causing Sb fractions to be redistributed, with EXC and CARB fractions increasing and other fractions decreasing [7,49,50,54]. In S1-BC, the Sb fractions were altered with rice growth. The EXC fraction surged from 2.20% to 2.81%, a 28.10% increase, with the addition of BC increasing the pH and DOC content and decreasing Eh values [49,50]; DOC derived from BC with abundant functional groups can interact with Sb, increasing the EXC fraction [51,52]. The CARB fraction rose from 6.63% to 10.66%, a 60.72% increase, mainly due to the BC’s ability to sustain a high-pH state which boosted carbonate production [7]. The Fe-Mn fraction decreased from 9.85% to 7.15%, a 27.34% reduction, possibly due to a fall in pH and Eh, which led to Fe-Mn oxides’ dissolution [49,55]. The OM fraction followed a similar trajectory to SOM, decreasing from 8.11% to 6.85%, a 15.48% reduction, with SOM decomposition being the primary cause [7]. Throughout the rice growth cycle, RES showed no apparent changes. The redistribution of Sb’s fractions affects its bioavailability and mobility, influencing Sb migration in the rhizosphere–rice system [24,38].

### 3.5. Sb Content in Rice Plants

**Figure 4:**
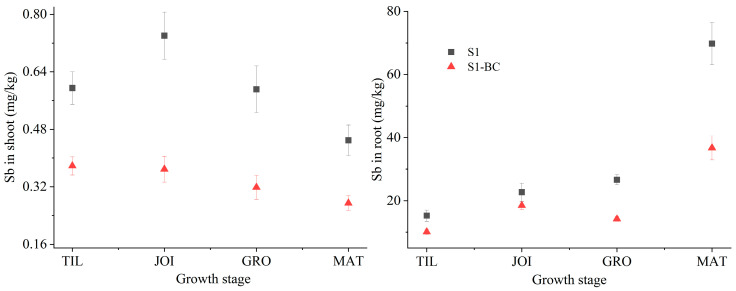
Sb levels in rice shoots and roots at four growth stages (S1: soil; S1-BC: soil with biochar).

Sb is a highly toxic element that can quickly accumulate in rice plants, constituting a latent risk to human health [56]. Figure 4 depicts the uptake of Sb by rice plants, revealing that the Sb content in rice plants varies with the rice growth stage and is influenced by BC.

The Sb concentration in the shoots decreased from 0.74 mg/kg to 0.45 mg/kg from the TIL to MAT stages of rice. This might be due to the increased shoot biomass, which generated dilution effects and Sb translocation to rice grains. The Sb in the roots increased slowly from 15.26 mg/kg to 26.64 mg/kg during the early growth stages from the TIL to GRF stages and then rose to 69.79 mg/kg at the MAT stage, owing primarily to the rapid increase in weight at the early rice growth stage, but with no discernible change at the late rice growth stage. A prior study by Meng et al. demonstrated that the biomass of rice shoots and roots varied with rice growth [57]. The accumulation of Sb in the roots and shoots positively affected the bioavailability and EXC fraction of Sb throughout rice growth. However, adding BC reduced Sb in the roots and shoots by 38.00–48.97% and 30.57–47.42%, respectively. In soil, biochar can interact with minerals to form organo-mineral complexes, and enhance iron plaque formation, increasing the binding sites of Sb and enhancing the rhizospheric effects to prevent Sb from migrating from the soil to the rice plant [17,58,59]. Biochar addition enhanced soil pH and bioavailable Sb and increased DOC with abundant functional groups that interact with Sb, which increased Sb migration from the shoots to grains, causing the Sb concentration in rice grains to rise slightly from 0.35 mg/kg to 0.38 mg/kg [60]. Total Sb in rice plants was calculated using Sb content and rice biomass, which suggested that biochar decreased Sb uptake by rice and affected rice biomass, as reported by Meng et al. [57]. Thus, biochar reduced Sb migration from the soil to rice plants while increasing Sb migration to rice grains.

**Figure 5:**
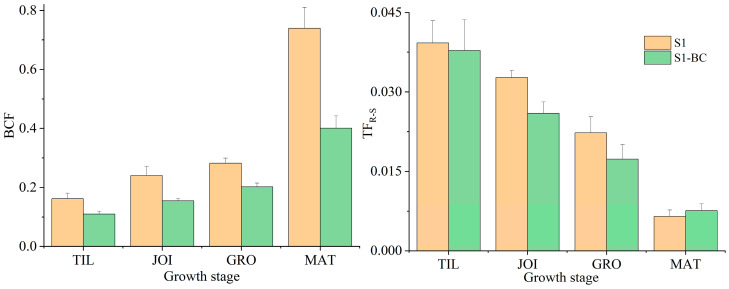
The BCF and TF of Sb during rice growth (S1: soil; S1-BC: biochar-amended soil).

The BCF and TF are the most frequently used transfer factors for evaluating the mobility of Sb from the soil to the plant and assessing the potential risk of Sb to human health. The BCF and TF of Sb varied with the growth stage, as shown in Figure 5. The BCF of Sb in S1 ranged from 0.16 to 0.74, while in S1-BC, it ranged from 0.11 to 0.40, corresponding to a reduction of 28.42–45.79% by BC. This is primarily because biochar, with its abundant functional groups, interacts with Sb and improves the rhizosphere environment to inhibit Sb migration from the soil to the rice roots [17,58,61]. The migration of Sb from the roots to the shoot of a plant is a crucial process for determining the potential risks to human health through the food chain. The TF_R-S_ values in S1 decreased from 39.2 × 10^−4^ to 6.5 × 10^−4^ as rice growth progressed from the TIL to MAT stages, indicating a reduced migration ability of Sb from the roots to shoots. Biochar addition lowered the TF_R-S_ values of Sb from 37.8 × 10^−4^ to 7.6 × 10^−4^, indicating decreased migration ability of Sb from rice roots to shoots. In other words, adding biochar enhanced Sb retention in the roots. A study by Xu et al. reported a similar result, where biochar increased As retention in roots [62]. The TF_S-G_ value was 0.83 for S1 and 1.38 for S1-BC, suggesting that biochar facilitated Sb migration from the shoots to grains, primarily due to the higher soil pH and DOC in S1-BC [60]. Thus, adding biochar increased Sb levels, endangering human health through food consumption.

## 4. Conclusions

This study demonstrated the redistribution of Sb within the rhizosphere–rice system relative to EFs (pH, Eh, SOM, and DOC), bioavailable Sb, Sb fractions, and Sb in rice plants during various rice growth stages. We also evaluated the functions of biochar. Sb redistribution within the rhizosphere–rice system changed dynamically during different growth stages and was influenced by biochar addition. Adding biochar altered the rhizosphere environments, increasing Sb mobility and decreasing Sb accumulation in the shoots and roots while boosting Sb accumulation in rice grains. However, using biochar as an amendment to remediate HM-contaminated soils requires further evaluation of its effect on Sb redistribution in the rhizosphere–rice system, an estimate of the potential harm to the food chain, and an assurance of its safe application. Despite these findings, there are limitations to this study, including the absence of a broader range of influence factors that could influence Sb redistribution in the rhizosphere–rice system, such as microbes and the transformation between Sb(III) and Sb(V). Future research should address these limitations to improve our understanding of biochar’s role in soil remediation and its impact on food safety.

## Data Availability

Data will be made available on request.

## Supplementary Materials

The following supporting information can be downloaded at https://www.mdpi.com/article/10.3390/toxics13050389/s1: Figure S1: The SEM (a) and FTIR spectrum (b) of BC; Figure S2: Soil moisture content in rice growth stages.
