# Supplementary figures and images for: Biochar Influences the Transformation and Translocation of Antimony in the Rhizosphere–Rice System
Source: Toxics. 2025 May 13;13(5):389. doi: 10.3390/toxics13050389 (PMC12115445; doi:10.3390/toxics13050389)

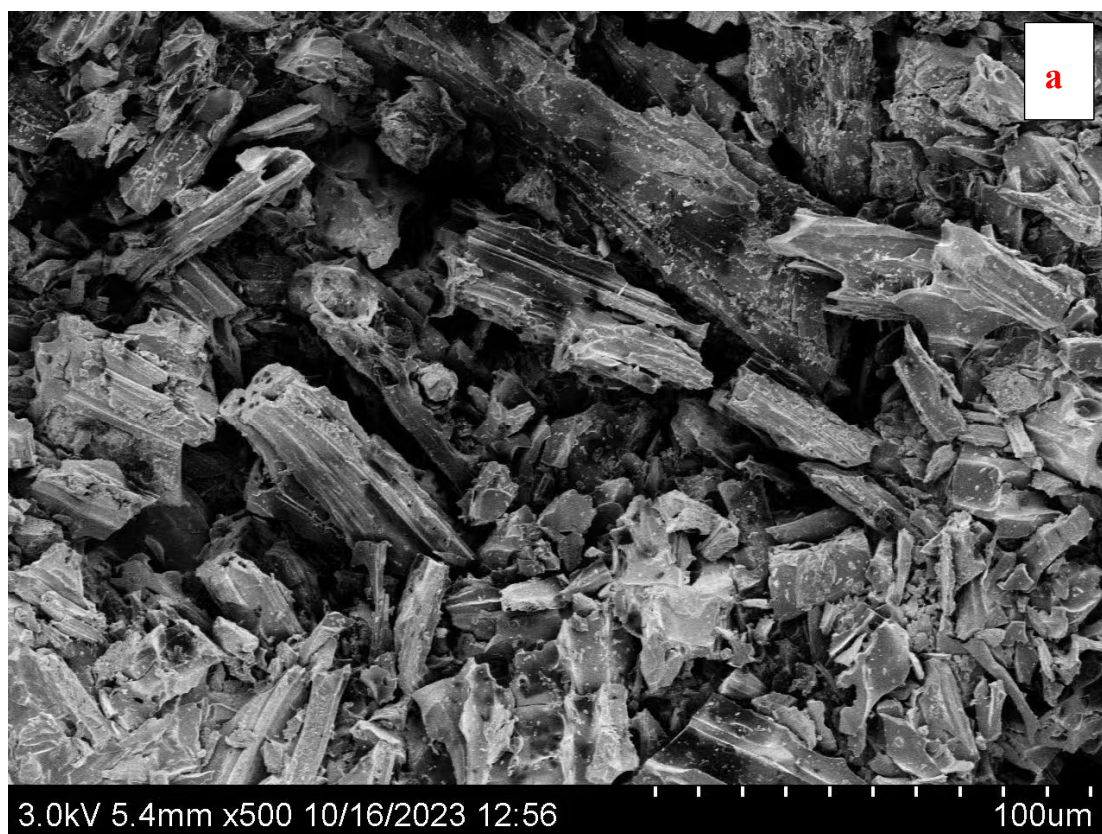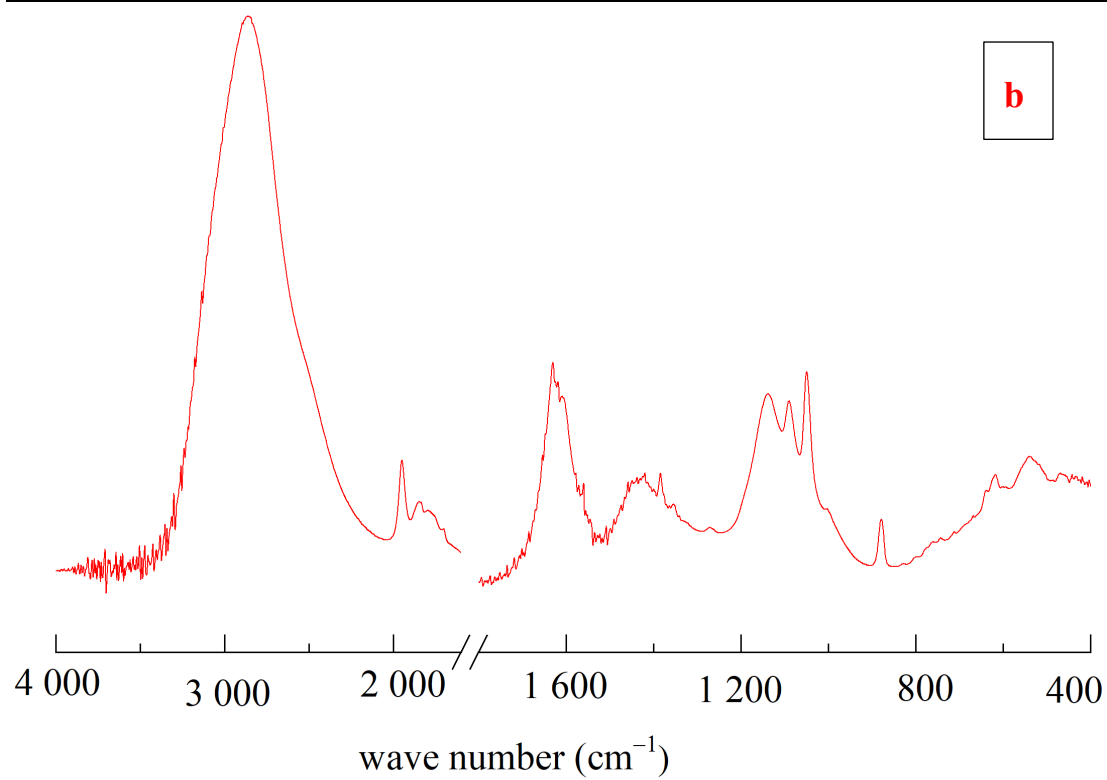

Figure S1 The SEM (a) and FTIR spectrum (b) of BC.

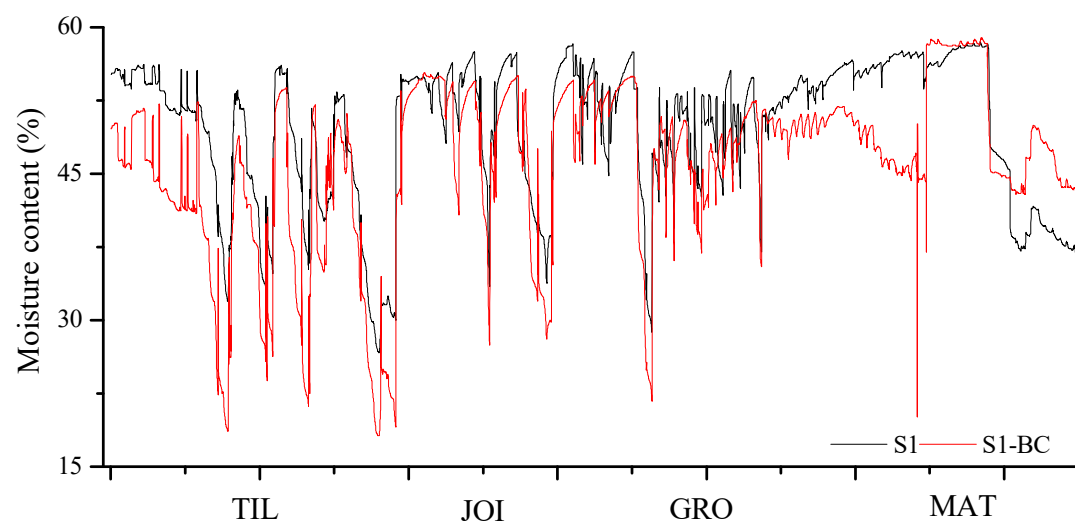

Figure S2 Soil moisture content in rice growth stages

Supplement: Supplementary file 1 [file toxics-13-00389-s001.zip › toxics-3600047-supplementary.pdf]
